# Bias in odds ratios by logistic regression modelling and sample size

**DOI:** 10.1186/1471-2288-9-56

**Published:** 2009-07-27

**Authors:** Szilard Nemes, Junmei Miao Jonasson, Anna Genell, Gunnar Steineck

**Affiliations:** 1 Division of Clinical Cancer Epidemiology, Department of Oncology, Sahlgrenska Academy, University of Gothenburg, Sweden; 2 Division of Clinical Cancer Epidemiology, Department of Oncology and Pathology, Karolinska Institutet, Sweden

## Abstract

**Background:**

In epidemiological studies researchers use logistic regression as an analytical tool to study the association of a binary outcome to a set of possible exposures.

**Methods:**

Using a simulation study we illustrate how the analytically derived bias of odds ratios modelling in logistic regression varies as a function of the sample size.

**Results:**

Logistic regression overestimates odds ratios in studies with small to moderate samples size. The small sample size induced bias is a systematic one, bias away from null. Regression coefficient estimates shifts away from zero, odds ratios from one.

**Conclusion:**

If several small studies are pooled without consideration of the bias introduced by the inherent mathematical properties of the logistic regression model, researchers may be mislead to erroneous interpretation of the results.

## Background

Logistic regression models yields odds-ratio estimations and allow adjustment for confounders. With a representative random sample from the targeted study population we know that odds ratio reflects the incidence ratio between the exposed and unexposed and we assume logistic regression models odd ratio without bias.

Decreased validity of the effect measure in epidemiological studies can be regarded as introduced in four hierarchical steps – confounding, misrepresentation, misclassification and analytical alteration of the effect measure [1].

Inherent mathematical properties in a model used may bias an effect measure such as an odds ratio modelled by logistic regression.

Logistic regression analyses have analytically attractive proprieties. As the sample size increases, the distribution function of the odds ratio converges to a normal distribution centered on the estimated effect. The log transformed odds ratio, the estimated regression coefficients, converges more rapidly to normal distribution [2]. However, as we will show below, especially for small studies, logistic models yields biased odds ratio.

Analytically derived bias causation can be traced back to the method of finding the point estimator. Logistic regression operates with maximum likelihood estimators. Odds ratios and beta coefficients both estimate the effect of an exposure on the outcome, the later one being the natural logarithm of the former one. For illustrative purposes, here we use beta coefficients instead of odds ratios but conclusions drawn stands for odds ratios as for beta coefficients.

The asymptotic bias of a maximum likelihood estimator, *bias*(*β*), can be summarized as

where *b*_*i*_(*β*) depends on the estimated beta coefficient, *β*. From this point of view bias is an additive term that depends on sample size *n *(or some other measure of information rate). Researchers aim to remove of the first order term, *O(n^-1^)*, namely the first term of the aforementioned equation.

## Methods

With help of the following simulation study we demonstrate how the sample size determines the size of bias in logistic regression parameter estimates. Assume an illness caused by one continuous exposure (e.g. BMI) and one discrete exposure variable (smoking, yes or no). The targeted population consists of 100000 individuals. The population parameter value for the continuous and discrete exposure variable is 2 and -0.9, respectively [see Additional file 1 for further details]. From this targeted population the researches randomly draw a sample with size determined by circumstances and resource limitations. Here we draw repeated samples with *a priori *determined sample sizes that varied from 100 to 1500 with increment 5. For each sample size we draw 1000 samples to assure a robust estimation. Then we fitted an ordinary least squares regression model to estimate *b*_1_(*β*). We estimated the relationship between *n*^-1 ^and the logistic regression coefficients for the given sample size by fitting the following equation based on the additive definition of the bias

As the sample size increases, *n *→ ∞, the bias converges to zero (lim_*n*→∞ _*b*_1_(*β*)*n*^-1 ^= 0), thus the intercept corresponds to unbiased estimate of the population parameter value. As an external validating measure we compared the estimated parametric curve with nonparametric estimation of the regression function and calculated its derivatives with kernel regression estimators and automatically adapted local plug-in bandwidth function. The derivatives were used as an empirical validation to our conclusions about the convergence rate.

## Results and discussion

**Figure 1:**
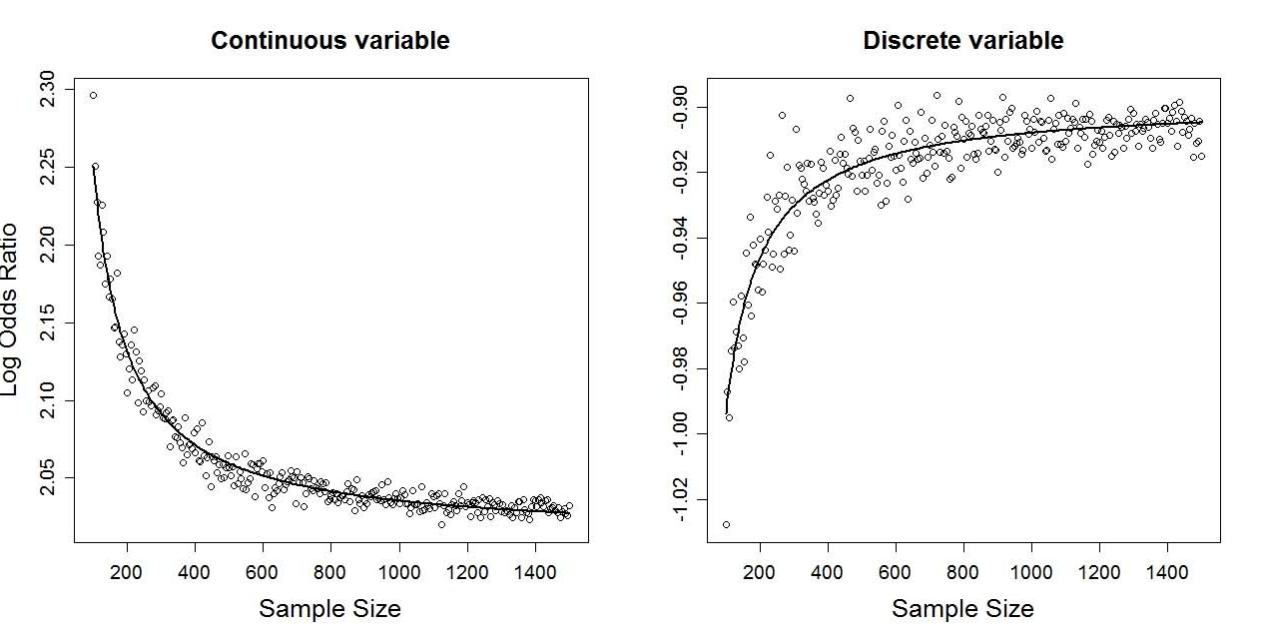
**Coefficient estimates and its sample size dependent systematic bias in logistic regression estimates**. The deviance from the true population value (2 respectively -0.9 in this case) represents the analytically induced bias in regression estimates.

**Figure 2:**
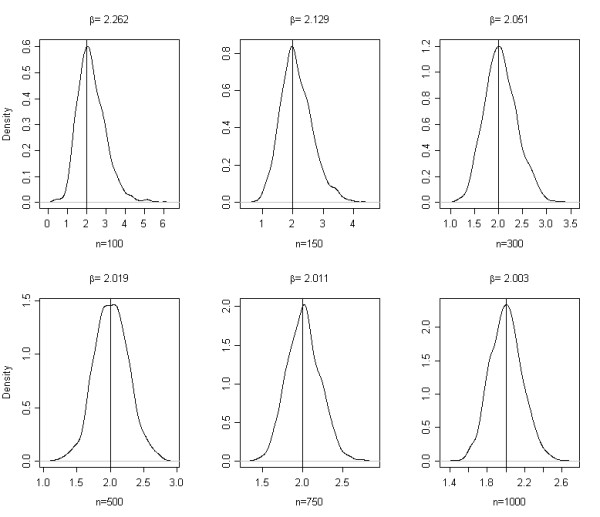
**Sampling distribution of logistic regression coefficient estimates at different sample sizes**.

**Figure 3:**
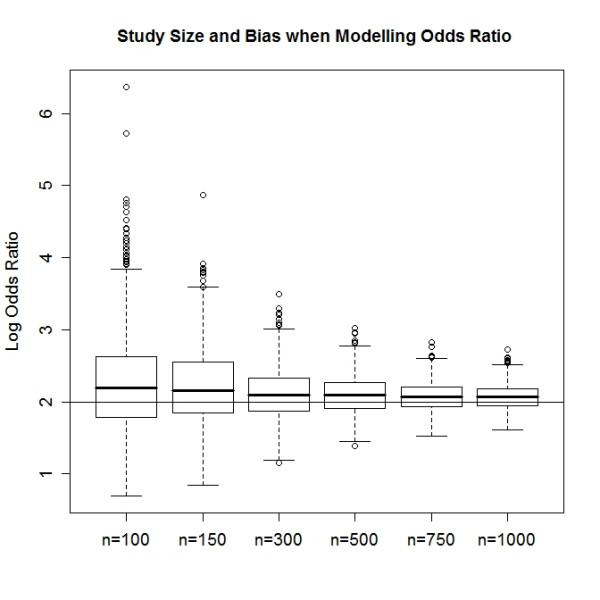
**Increasing sample size not only reduces the analytically induced bias in regression estimates but protects against extreme value estimates**.

Table 1 summarizes the estimated empirical bias in estimated regression coefficients. With increasing sample size the estimated coefficients asymptotically approaches the population value (Figure 1). The fit is better for continuous variables (R^2 ^= 0.963) than for discrete one (R^2 ^= 0.836). This translates to a greater variability in logistic regression estimates for discrete variables. For both the continuous and discrete exposure variables the asymptotic bias converges to zero as the sample size increase, but the convergence intensity differs. Also the sampling density function is rather skewed in smaller samples and approaches to a symmetric distribution with increasing sample size (Figure. 2). Skewed sampling distribution more frequently result in extreme value estimates, the proportion of which decreases with increasing sample sizes (Figure 3).

**Table 1:** Empirical Estimation of the Magnitude of the Asymptotic Bias of Logistic Regression Coefficients.

	Estimate	SE	t-value	Pr(>|t|)
	Continuous variable			
	
Intercept	2.011	0.00072	2785.9	<0.0001
*n*^-1^	23.9	0.276	86.48	<0.0001
	
	Discrete variable			
	
Intercept	-0.898	0.00065	-1369.34	<0.0001
*n*^-1^	-9.524	0.251	-37.92	<0.0001

Thus we can conclude that studies employing logistic regression as analytical tool to study the association of exposure variables and the outcome overestimate the effect in studies with small to moderate samples size. The magnitude of this analytically derived bias depends on the sample size and on the data structure. The small sample size induced bias is a systematic one, bias away from null. Regression coefficient estimates shifts away from zero, odds ratios from one. This analytic bias is an acknowledged statistical phenomenon [3-8], but partly is unknown among practitioners and partly ignored. Justification for the ignorance lies in the assumption that the bias is much smaller than the estimate's standard error [9]. Consistent estimators can be biased in finite samples and corrective measures are required. However, caution is advised as bias correction might inflate the variance and mean squared error of an estimate [10]. Several corrective measures have been suggested in the literature; like the bias corrected estimate  or the jackknife [4]. Bootstrapping, especially the quadratic bootstrap method, have proved to be a feasible corrective measure [11]. Jewell proposes alternatives to the maximum likelihood estimator, but concludes that the slight gain in precision might not be worth the increased complexity [5]. Bias-corrected maximum likelihood estimates can be obtained with the help of supplementary weighted regression [7] or by suitable modification of the score function [3]. A proper and well designed sampling strategy can improve the small sample performance of the estimate [12].

Studies conducted on the same topic with varying sample sizes will have varying effect estimates with more pronounced estimates in small sample studies, or studies with highly stratified data. In small or even in moderately large sample sizes their distributions are highly skewed and odds ratios are overestimated. Here we can't give strict guidelines about how large an adequate sample should be this is largely study specific. Long [13] states that it is risky to use maximum likelihood estimates in samples under 100 while samples above 500 should be adequate. However this varies greatly with the data structure at the hand. Studies with very common or extremely rare outcome generally require larger samples. The number of exposure variables and their characteristics strongly influences the required sample size. Discrete exposures generally necessitate larger sample sizes than continuous exposures. Highly correlated exposures need larger samples as well.

Small study effect, the phenomenon of small studies reporting larger effects than large studies, repeatedly has been described [14]. A selective publication of "positive studies" may partly explain this phenomenon. We have however illustrated that odds ratios are overestimated in small samples due to the inherent properties of logistic regression models. This bias might in a single study not have any relevance for the interpretation of the results since it is much lower than the standard error of the estimate. But if a number of small studies with systematically overestimated effect sizes are pooled together without consideration of this effect we may misinterpret evidence in the literature for an effect when in the reality such does not exist.

## Conclusion

Studies with small to moderate samples size employing logistic regression overestimate the effect measure. We advice caution when small studies with systematically overestimated effect sizes are pooled together.

## Competing interests

The authors declare that they have no competing interests.

## Authors' contributions

NSz conceived the study and participated in its design, carried out its implementing and drafted the first version of the manuscript. JMJ participated in study design. AG participated in study implementation. GS coordinated the study. All authors contributed to the writing and approved the final version.

## Pre-publication history

The pre-publication history for this paper can be accessed here:

http://www.biomedcentral.com/1471-2288/9/56/prepub

## Supplementary Material

Additional file 1 **Bias in odds ratios by logistic regression modelling and sample size**. Detailed description of the study design Click here for file

## References

[B1] Steineck G Hunt H Adolfsson J A hierarchical step-model of bias – Evaluating cancer treatment with epidemiological Methods Acta Oncologica 2006 45 421 429 10.1080/02841860600649293 16760178

[B2] Agresti A Categorical Data Analysis 1990 Wiley Series in Probability and Statistics, New Jersey, John Wiley & Sons Inc

[B3] Firth D Bias reduction of maximum likelihood estimates Biometrica 1993 80 1 27 38 10.1093/biomet/80.1.27

[B4] Cox DR Hinkley DV Theoretical Statistics 1982 Chapman and Hall, London

[B5] Jewell NP Small-sample bias of point estimators of the odds ratio from matched sets Biometrics 1984 40 412 435 10.2307/2531395 6487726

[B6] Ejigou A Small-sample properties of odds ratio estimators under multiple matching in case-control studies Biometrics 1990 46 61 69 10.2307/2531630

[B7] Corderio GM McCullagh P Bias correction in Generalized Linear Models JR Statist Soc B 1991 53 3 629 643 http://www.jstor.org/pss/2345592

[B8] Nam JM Bias-corrected maximum likelihood estimator of a log common odds ratio Biometrica 1993 80 3 688 694 10.1093/biomet/80.3.688

[B9] Pawitan Y In all Likelihood: Statistical Modelling and Inference Using Likelihood 2001 Oxford University Press, New York

[B10] MacKinnon JG Smith AA Jr Approximate bias correction in econometrics Journal of Econometrics 1998 85 2 205 230 10.1016/S0304-4076(97)00099-7

[B11] Claeskens G Aerts M Molenberghs G A quadratic bootstrap method and improved estimation in logistic regression Statistics & Probability Letters 2003 61 383 394 10.1016/S0167-7152(02)00397-8

[B12] Deitrich J The effects of sampling strategies on the small sample properties of the logit estimator Journal of Applied Statistics 2005 32 543 554 10.1080/02664760500078888

[B13] Long SL Regression Models for Categorical and Limited Dependent Variables 1997 Advanced Quantitative Techniques in the Social Sciences 7. SAGE Publications, Thousand Oak

[B14] Sterne JAC Gavaghan D Egger M Publication and related bias in meta-analysis: Power of statistical tests and prevalence in the literature Journal of Clinical Epidemiology 2000 53 1119 1129 10.1016/S0895-4356(00)00242-0 11106885

