# Supplementary material for: Bias in odds ratios by logistic regression modelling and sample size
Source: BMC Med Res Methodol. 2009 Jul 27;9:56. doi: 10.1186/1471-2288-9-56 (PMC2724427; doi:10.1186/1471-2288-9-56)
Supplement: Additional file 1 — Bias in odds ratios by logistic regression modelling and sample size. Detailed description of the study design [file 1471-2288-9-56-S1.pdf]

Detailed description of the study design

1. Generate the targeted study population.

The data set has one binary response variable (y) and one continuous (x) and one discrete (z) exposure variable. The continuous exposure variable is normally distributed with mean 2 and a standard deviation of 0.7 (R code: `rnorm(100000, 2, 0.7)`). To better mirror a real life scenario with correlating predictor variables we conditioned the discrete variable on the continuous one. If the continuous variable exceeds 2.5 (P(x>2.5) =0.2375) then the discrete exposure variable has Bernoulli distribution with p=0.6 otherwise p=0.4. This created a week dependency structure, an association between the exposures. (R code: `ifelse(x>2.5, rbinom(100000, 1, 0.6), rbinom(100000, 1, 0.4))`).

We assumed the following additive effect of the exposure variables $\theta = 0.1 + 2x - 0.9z$. The model was used to generate the outcome, using the logistic distribution. We estimated the probability that response variable takes the value 1 as follow $P(y = 1) = \frac{e^{\theta}}{1+e^{\theta}}$. To impose the natural variability we generated a uniformly distributed variable (u) bounded between 0 and 1. If $P(y=1)<u$ the we assigned value 1 to the outcome otherwise zero (R code: `ifelse(runif(100000) < plogis(theta), 1, 0)).`

Note: for a vividly descriptive but somewhat more complicated example one should consult the “Design” library of R.

2. Take samples from the target population

Each observation (each row) in the simulated data set is associated with an index- the number of the row. Thus we have an index that spans from 1 to 100000. We randomly sampled numbers from this index with size of the desired sample. From the data base we then extracted the rows with the sampled index values.

Given a data frame called "`simdata`" with 100000 observations and a desired sample of size 500 the following R code could be a viable option: `simdata[sample(100000, 500),]`.
